# Emerging trends and research foci in gastrointestinal microbiome

**DOI:** 10.1186/s12967-019-1810-x

**Published:** 2019-02-28

**Authors:** Xiaoquan Huang, Xiaowen Fan, Jun Ying, Shiyao Chen

**Affiliations:** 1 Department of Gastroenterology and Hepatology, Zhongshan Hospital, Fudan University, 180 Fenglin Road, Shanghai, 200032 China; 2 Center of Evidence-Based Medicine, Fudan University, 180 Fenglin Road, Shanghai, 200032 China; 3 Mount Sinai St Luke’s and Mount Sinai West, New York, NY 10019 USA; 4 Fudan University Library, Fudan University, Shanghai, 200032 China

**Keywords:** Gastrointestinal microbiome, Research foci, Dysbiosis, Co-citation analysis

## Abstract

**Background:**

Gastrointestinal microbiome has drawn an increasing amount of attention over the past decades. There is emerging evidence that the gut flora plays a major role in the pathogenesis of certain diseases. We aimed to analyze the evolution of gastrointestinal microbiome research and evaluate publications qualitatively and quantitatively.

**Methods:**

We obtained a record of 2891 manuscripts published between 1998 and 2018 from the Web of Science Core Collection (WoSCC) of Thomson Reuters; this record was obtained on June 23, 2018. The WoSCC is the most frequently used source of scientific information. We used the term “Gastrointestinal Microbiomes” and all of its hyponyms to retrieve the record, and restricted the subjects to gastroenterology and hepatology. We then derived a clustered network from 70,169 references that were cited by the 2891 manuscripts, and identified 676 top co-cited articles. Next, we used the bibliometric method, CiteSpace V, and VOSviewer 1.6.8 to identify top authors, journals, institutions, countries, keywords, co-cited articles, and trends.

**Results:**

We identified that the number of publications on gastrointestinal microbiome is increasing over time. 112 journals published articles on gastrointestinal microbiome. The United States of America was the leading country for publications, and the leading institution was the University of North Carolina. Co-cited reference analysis revealed the top landmark articles in the field. Gut microbiota, inflammatory bowel disease (IBD), probiotics, irritable bowel disease, and obesity are some of the high frequency keywords in co-occurrence cluster analysis and co-cited reference cluster analysis; indicating gut microbiota and related digestive diseases remain the hotspots in gut microbiome research. Burst detection analysis of top keywords showed that bile acid, obesity, and *Akkermansia muciniphila* were the new research foci.

**Conclusions:**

This study revealed that our understanding of the link between gastrointestinal microbiome and associated diseases has evolved dramatically over time. The emerging new therapeutic targets in gut microbiota would be the foci of future research.

## Background

The human gastrointestinal flora is an essential organ that plays an important role in gastrointestinal and overall health. Understanding of this organ has evolved significantly over the past decades due to the large quantity of impactful research on the gastrointestinal microbiome.

Since the 1990s, the development of culture independent molecular methods, including 16S ribosomal RNA (16S rRNA) gene sequencing and metagenomic sequencing methods, has allowed for quantitative analysis of the composites of gut microbiome and provided a better understanding of its variation and function. 16S rRNA genes are bacteria’s small subunit molecules that include both conserved and variable regions that allow designing probes or primers to detect and identify bacteria, and specify a phylum, a group, a genus, or even a species [1]. This technique has increased previous culture-based estimates of 200–300 colonic species to 15,000–36,000 individual species [2]. Metagenomic sequencing represents a powerful alternative to rRNA sequencing, it utilizes taxonomically informative gene tags to target and amplify genomes of interest, analysis of this data allows researchers to determine the composite and function of different microbiomes, it is widely used for global characterization of the genetic potential of ecologically complex environments [3, 4].

The human metagenome is composed of Homo sapiens genes and genes present in the genome of the microbes that colonize the human body, estimates have the latter as encoding 100-fold more unique genes than the human genome [5, 6]. The majority of the colonizing microbes reside in the gut. Previous study revealed that there is a functional core conserved in each individual, which represents the full minimal human gut metagenome that is required for the proper functioning of the gut ecosystem. The human gut ecosystem includes important beneficial functions, such as: fermentation of dietary fibers into short-chain fatty acid (SCFA) which counts up to 10% of the human energy source, degradation of complex polysaccharides, and synthesis of indispensable vitamins and amino acids [4, 6–8]. The gut flora also produce multiple metabolites that function in protecting epithelial lining integrity, stimulating intestinal angiogenesis, and regulating immune response [9–11]. These reflect that gut flora are not just commensal with human hosts, there is mutualism in the relationship between them.

Sampling is a key step for studies on gut microbiome. A significant number of studies are human based, while most others are mice based. Fewer studies were performed on gut microbiota from tissue samples, while most others were performed on fecal microbiota. In different regions of the gastrointestinal tract, the composition and luminal concentrations of dominant microbial species vary; the distal ileum, cecum, and colon are the most common sites for tissue sample harvesting due to high quantity and variety of gut flora in these regions [3, 12, 13]. Fecal samples are often used to investigate mucosa-associated microbiota because they are easy to collect, noninvasive, are approved to be a reproducible and relevant source of biomarkers [1]. But there is still concern that the fecal microbiota may represent a combination of shed mucosal bacteria and a separate nonadherent luminal population, thus making the result less reliable [13].

Over 98% of the gut microbiota is composed of four phyla of bacteria: Firmicutes, Bacteroidetes, Proteobacteria, and Actinobacteria. The dominant majority are from either Firmicutes or Bacteroidetes [2, 13]. Gut microbiota composition varies based on sampling region. Research has revealed that the Bacillus subgroup of Firmicutes and Actinobacteria are more prevalent in the small intestine, while Bacteroidetes and Lachnospiraceae are more prevalent in the colon [2]. Human gut microbiome is more similar among family members than unrelated individuals; intra-personal differences are minor compared to inter-personal differences in gut microbiota, indicating short term environmental change does not play a major part in microbiota composition [14].

The proper interaction between host gut mucosa and gut microbiota is important in maintaining mucosal homeostasis. The gut microbiota is proposed to shape host immunity and host immunity functions via secreting molecules that protect the mucosal barrier integrity, proper secretion of luminal antimicrobial peptides and immunoglobulins, down-regulation of innate and adaptive immune responses to commensal bacteria, and elimination of translocated bacteria across epithelial barrier [8, 11, 15]. Mucosal homeostasis is key to both host and gut flora and can be disrupted in dysbiosis causing chronic intestinal inflammation.

CiteSpace is an application designed to generate and analyze networks of co-cited references based on bibliographic database [16]. We employed this method to analyze trends and hotspots of global publications on gastrointestinal microbiome between 1998 and 2018.

## Methods

We obtained a record of 2891 manuscripts published between 1998 and 2018 from the Web of Science Core Collection (WoSCC) of Thomson Reuters; this record was obtained on June 23, 2018. The WoSCC is the most frequently used source of scientific information. We used the term “Gastrointestinal Microbiomes” and all of its hyponyms to retrieve the record, and restricted the subjects to gastroenterology and hepatology. We then derived a clustered network from 70,169 references that were cited by the 2891 manuscripts, and identified 676 top co-cited articles. Next, we used the bibliometric method, CiteSpace V, and VOSviewer 1.6.8 to identify top authors, journals, institutions, countries, keywords, co-cited articles, and trends.

## Results

### Distribution of articles by publication years

**Fig. 1:**
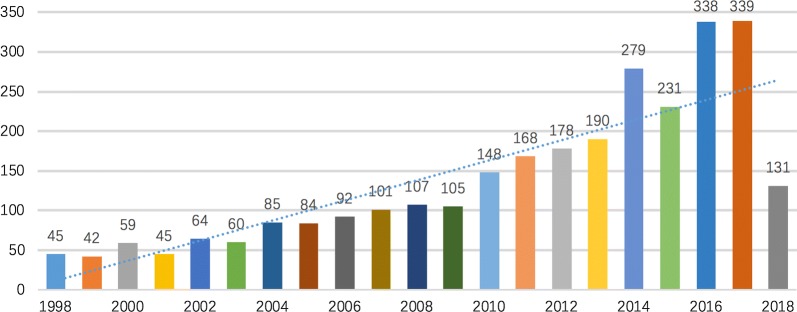
Trends in the number of scholarly publications on gastrointestinal microbiome research from 1998 to 2018

Between 1998 and 2018, 2891 original research articles were published. There was an increasing trend for quantity of research publications on gastrointestinal microbiome (Fig. 1). The number of published articles on gastrointestinal microbiome steadily increased from 1998 through 2009, and then the number of publications significantly increased from 2010 onwards, with the number of publications almost doubled in 2014 compared to 2010. During 2016 through 2017, the activity in gut microbiome research reached a peak.

### Journal analysis

The total number of journals that published the 2891 articles on gastrointestinal microbiome was 112. The characteristics of the 10 most active journals and the main ideas of their representative articles are shown in Table 1 [1, 17–25]. All the publishers of the journals are located in either the United States of America or the United Kingdom. Digestive Diseases and Sciences published the greatest number of articles on gut microbiome, followed by World Journal of Gastroenterology and Gut. Regarding impact factor, Gastroenterology has the highest impact factor, followed by Gut and Alimentary Pharmacology Therapeutics.

**Table 1:** Top 10 journals that published articles on gastrointestinal microbiome

Journal	Frequency	Country	JCR	Impact factor (2017)	Eigen factor score	Representative article and main ideas
Digestive Diseases and Sciences	248	USA	Q3	2.819	0.022940	*Saccharomyces boulardii* is important in the maintenance treatment of Crohn’s disease [17]
World Journal of Gastroenterology	229	USA	Q2	3.300	0.074030	Butyrate, short-chain fatty acid, has a high potential for a therapeutic use in human medicine [18]
Gut	213	UK	Q1	17.016	0.071930	The metagenomic approach revealed a reduced complexity of the bacterial phylum Firmicutes as a signature of Crohn’s disease [19]
Inflammatory Bowel Diseases	176	USA	Q1	4.347	0.033880	Low counts of *Faecalibacterium prausnitzii* are associated with a reduced protection of the gut mucosa in colitis [1]
Journal of Pediatric Gastroenterology and Nutrition	153	USA	Q3	2.752	0.018600	The 16S rRNA-based techniques provided more accurate quantitative data on gut flora development in newborns than conventional culture techniques [20]
Gastroenterology	152	USA	Q1	20.773	0.120200	New probiotic preparation is effective in preventing flare-ups of chronic pouchitis [21]
Journal of Clinical Gastroenterology	134	USA	Q3	2.968	0.010910	Gut microbiome can be reprogrammed to restore beneficial host structure and functions [22]
American Journal of Physiology Gastrointestinal and Liver Physiology	132	USA	Q2	3.293	0.018460	Increased intestinal permeability and portal endotoxemia contribute to the pathogenesis of nonalcoholic steatohepatitis [23]
Alimentary Pharmacology Therapeutics	96	UK	Q1	7.357	0.035700	The effects of alteration to the bacterial flora support the hypothesis of a pathophysiological role for the intestinal environment in ulcerative colitis [24]
Gut Pathogens	95	UK	Q3	2.809	0.001930	A significant rise in *Lactobacillus* and *Bifidobacteria* resulted in a decrease in anxiety symptoms gut–brain interface [25]

### Country and institution analysis

The 2891 articles on gastrointestinal microbiome research were published by research groups in 41 countries/regions. The top 10 countries (6 European countries, 2 Asian countries, and 2 North American countries) published 2692 articles, accounting for 93.12% of the total number of publications. The leading country was the United States, which took up 31.92% (923/2891) of the total, the next 2 high production countries were Italy and the People’s Republic of China, which took up 10% and 8% of the total, respectively. There were more than 370 research institutions that published articles related to gut microbiome. The leading research institution with the highest number of publications was the University of North Carolina, which had 64 articles with the strongest citation burst from 2003 to 2011, followed by Harvard University (53 articles), Mayo Clinic (41 articles), French National Institute for Agricultural Research (40 articles), and Massachusetts General Hospital (39 articles).

### Keyword co-occurrence cluster analysis of research hotspots

**Fig. 2:**
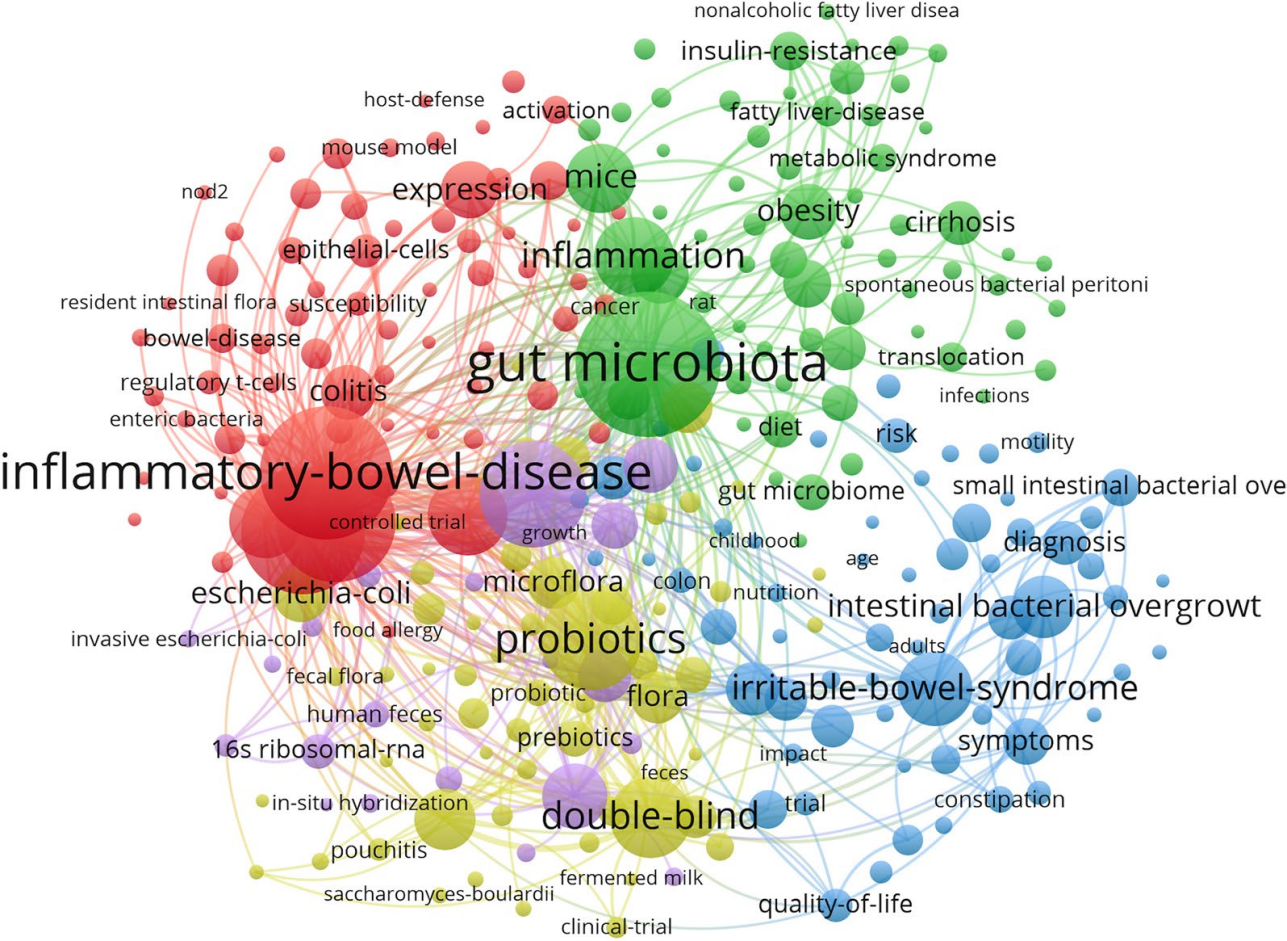
Map of keyword clustering showed 274 keywords with a minimum of 20 occurrence and divided into 5 clusters

VOSviewer keyword analysis of the 2891 articles identified 274 keywords with a minimum of 20 occurrences and divided them into 5 clusters (Gut microbiota, IBD, probiotics, double-blind, and irritable bowel syndrome) (Fig. 2).

### Top co-cited articles analysis

**Fig. 3:**
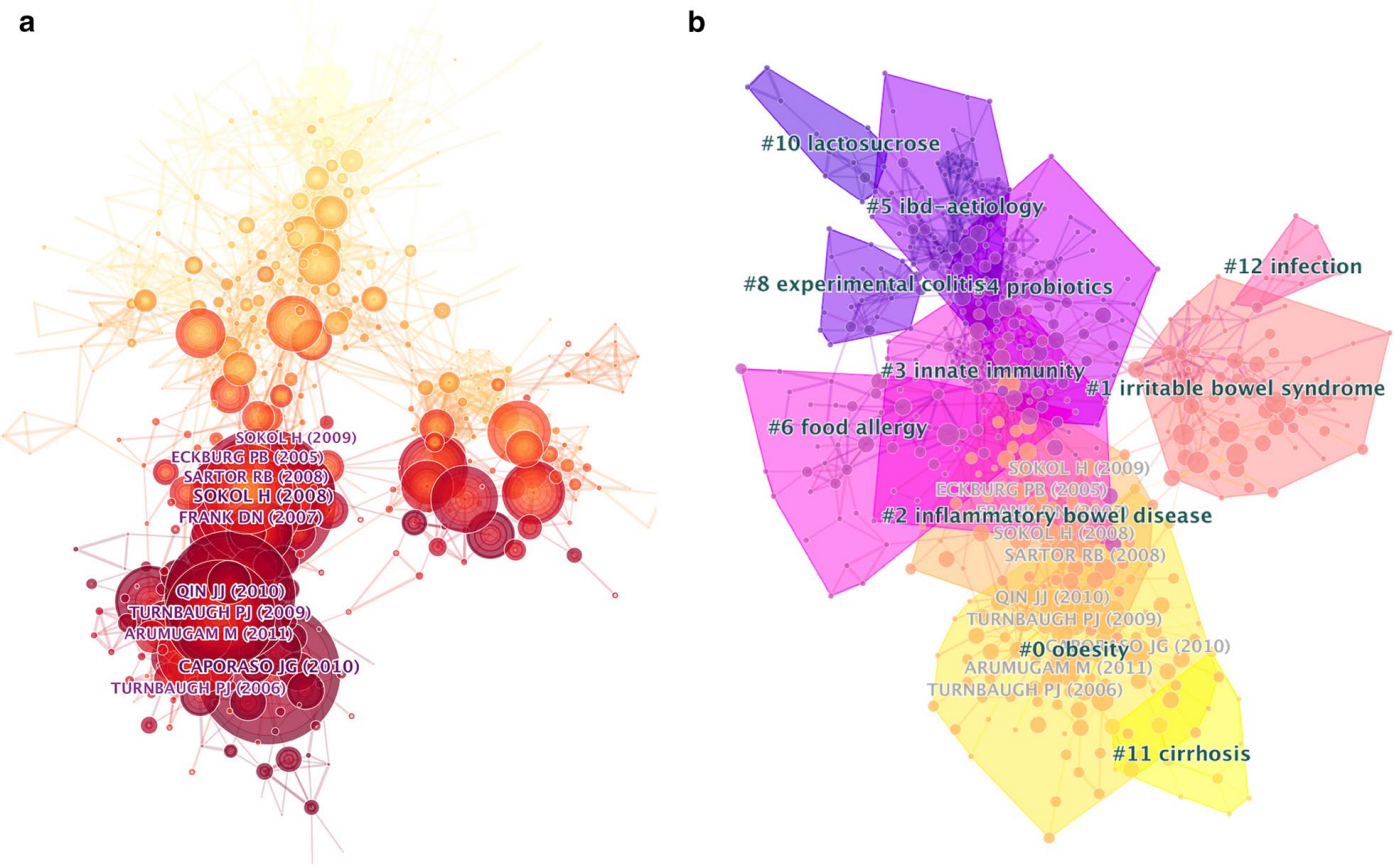
**a** Co-citation map of authors on clustered network of gut microbiome; **b** clustered network of co-cited articles on gut microbiome and their sub-networks

The clustered network is derived from 70,169 references (including duplicates) that were cited by the 2891 articles. The clustered network of gastrointestinal microbiome is demonstrated in this part. Citation reference knowledge maps consist of references with higher centrality and citation counts. Visualization of co-cited articles showed a total of 676 nodes and 1427 links (Fig. 3a). Each node represents a cited article. The area of each node is proportional to the total co-citation frequency of the associated article.

The top 10 co-cited articles, their cited frequency, and cited half-year life are shown in Table 2. Sokol [12] in PNAS had the highest number of citations (168 citations), followed by Caporaso [17] in Nature Methods (163 citations), and Qin [4] in Nature (148 citations). These articles are often considered fundamental in gastrointestinal microbiome research.

**Table 2:** The top 10 co-cited articles, cited authors and cited references

Authors	Years	Journal	Cited frequency	Half year	Title	Focus	Method
Sokol [12]	2008	PNAS	168	5	*Faecalibacterium prausnitzii* is an anti-inflammatory commensal bacterium identified by gut microbiota analysis of Crohn disease patients	Crohn disease	FISH
Caporaso [17]	2010	Nature Methods	163	6	QIIME allows analysis of high-throughput community sequencing data	Sequencing technology	Data analysis platform
Qin [4]	2010	Nature	148	5	A human gut microbial gene catalogue established by metagenomic sequencing	Gut microbiome and human health	Metagenomic sequencing
Turnbaugh [14]	2009	Nature	128	5	A core gut microbiome in obese and lean twins	Obesity	16s rRNA and Metagenomic sequence
Frank [2]	2007	PNAS	119	5	Molecular-phylogenetic characterization of microbial community imbalances in human inflammatory bowel diseases	Inflammatory bowel diseases	Broad-Range PCR Analysis
Sartor [8]	2008	Gastroenterology	113	5	Microbial influences in inflammatory bowel diseases	Inflammatory bowel diseases	Microbial alteration and immune response
Arumugam [56]	2011	Nature	107	3	Enterotypes of the human gut microbiome	Enterotypes	Metagenomics
Eckburg [13]	2005	Science	103	6	Diversity of the human intestinal microbial flora	Intestinal microbiome diversity	16s rRNA
Turnbaugh [3]	2006	Nature	92	6	An obesity-associated gut microbiome with increased capacity for energy harvest	Obesity	Metagenomic and biochemical analyses
Sokol [1]	2009	Inflammatory Bowel Diseases	90	5	Low counts of *Faecalibacterium prausnitzii* in colitis microbiota	Colitis	16s rRNA

Half year, the median age of the articles that were cited in the JCR (Journal Citation Reports) year

PNAS, Proceedings of the National Academy of Sciences of the United States of America

### Co-cited reference cluster analysis

**Fig. 4:**
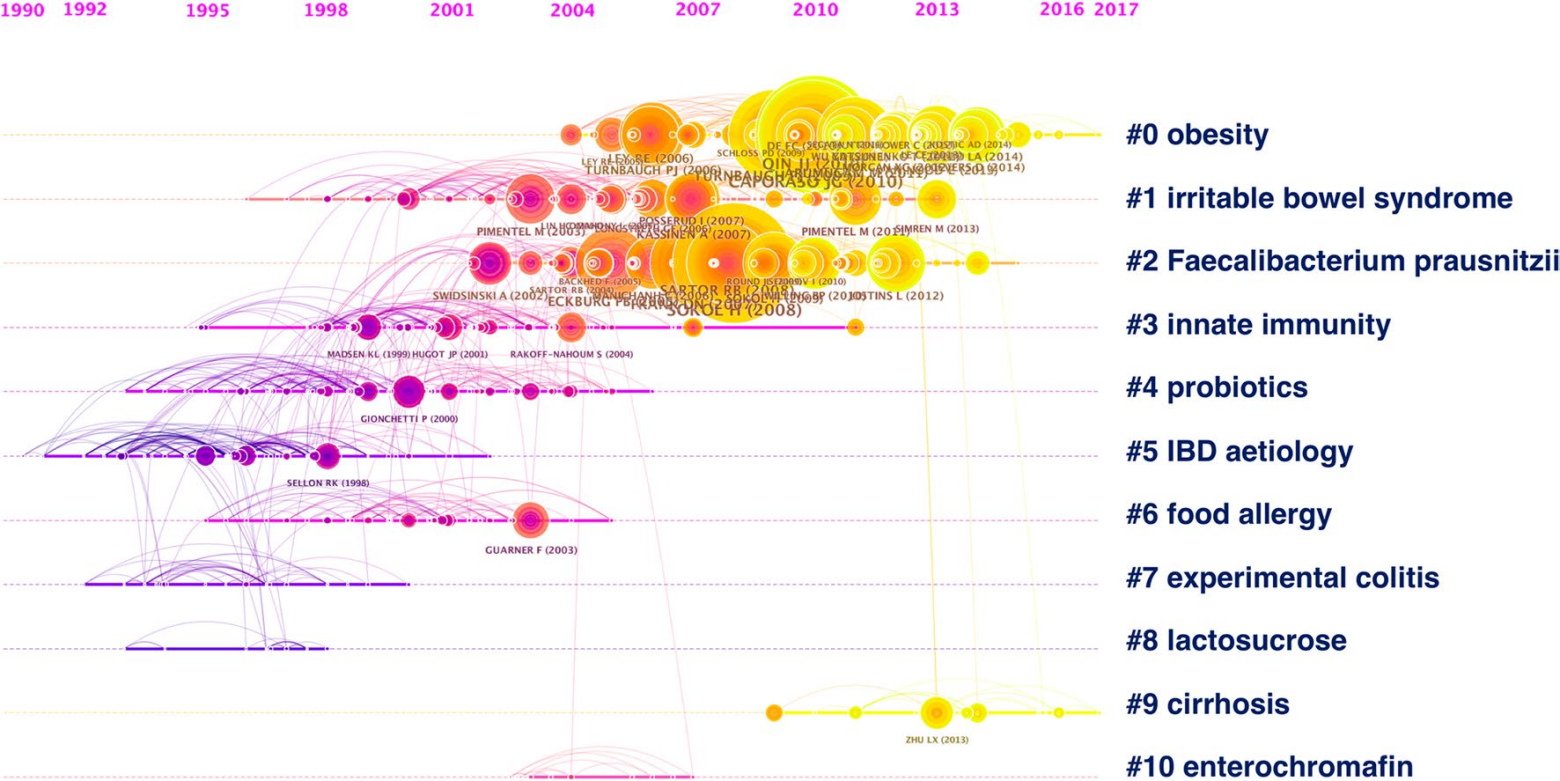
Timeline of co-citation clusters. Top clusters are labeled on the right and landmark articles are labeled

To detect research hotspots, we mapped the 676 top co-cited articles cited by 2891 original articles via a clustered network in hierarchical order (Fig. 3b). The nodes represent different cited references and the clusters represent a distinct specialty or a thematic concentration. The citation reference knowledge map consists of references with higher centrality and citation counts. The area of each node is proportional to the total co-citation frequency of the associated reference. The co-cited references could be clustered into eleven main sub-clusters including obesity, irritable bowel syndrome, IBD, innate immunity, probiotics, etc. Figure 4 displays a timeline visualization of distinct co-citation and shows that Cluster #0 obesity and Cluster #2 *Faecalibacterium prausnitzii* had the highest concentration of nodes with citation bursts, and research foci seems to have shifted from IBD to obesity and cirrhosis. This supports the finding of the emerging focus in gut microbiome.

### Burst detection

**Fig. 5:**
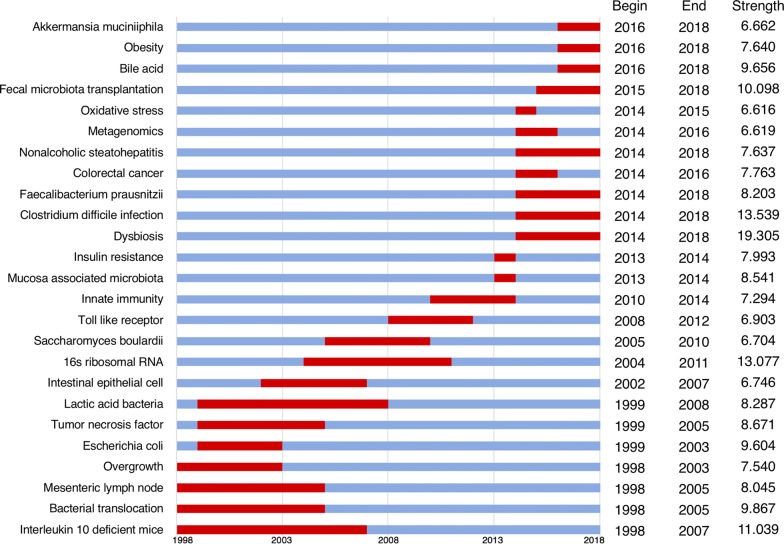
Keywords with the strongest citation bursts in articles on gastrointestinal microbiome published during 1998–2018

Burst detection identified articles that have attracted the attention of peer researchers. We detected bursts between 1998 through 2018 based on analysis of the 2891 original articles. The timeline is depicted as a blue line, and the time interval that a subject was found to have a burst is shown as a red segment on the blue timeline, indicating the beginning year, the ending year, and the duration of the burst. Among the top 191 keywords with the highest burst strength, we were particularly interested in those keywords with research significance which indicate the evolution trend of the gut microbiome (Fig. 5). During the entire time period from 1998 through 2018, dysbiosis had the highest burst strength, followed by Clostridium difficile infection, 16s rRNA, Interleukin 10 deficient mice, and fecal microbiota transplantation. Interleukin 10 deficient mice, bacterial translocation and overgrowth, and mesenteric lymph node became the research foci since 1998 and then the intestinal epithelial cell; 16s rRNA sequencing became a research focus since 2004, followed by toll like receptor and innate immunity, marking microbiome research entering the new era; regarding the bursts with most recent onset: bile acid, obesity, and *Akkermansia muciniphila* were the strongest bursts that started in 2016.

## Discussion

In this study, we utilized information visualization to analyze original articles on gut microbiome published from 1998 through 2018. Based on the trends we identified that showed an increasing number of scientific research publications over the 20-year period; we conclude that gut microbiome has become a subject of growing study and an increasingly important area of research. The burst of research activity after 2010 is likely due to the fact that some research publications that have had a major impact on the understanding of gut microbiome diversity and enterotype, sequencing data analysis in microbial communities, and related digestive diseases especially IBD, and obesity were published during 2005 and 2010 (Table 2); thus, triggering researchers’ attention in this field and causing an increased number of publications.

The journals with the greatest number of articles in gut microbiome are mostly major journals in gastroenterology including Digestive Diseases and Sciences, Gastroenterology, Gut, Inflammatory Bowel Diseases, World Journal of Gastroenterology, etc. This indicates that gut microbiome has become a central topic of gastroenterology research. Analysis of the co-citation map of authors and top cited authors (Fig. 3a and Table 2) during 1998 to 2018 showed that Sokol, Caporaso, Turnbaugh, Frank, Eckburg, and a few other authors are the researchers with publications that significantly impacted the research trend and current understanding of gut microbiome.

Among the top 10 co-cited articles, Caporaso et al. introduced QIIME (quantitative insights into microbial ecology), an open-source software that takes sequencing data to interpretation and database deposition, as a fundamental tool in analyzing microbial communities [26]. Eckburg et al. studied prokaryotic ribosomal RNA gene sequences from colonic mucosa tissue and feces of healthy subjects, identified the majority of bacterial phylotypes as novel or uncultivated species, and revealed significant inter-subject variability and differences between stool and adherent mucosa microbial composition, which is relevant to different sampling methods during microbiome studies [13]. Qin et al. established a catalogue of human intestinal microbial genes by using extensive illumina-based sequencing of total faecal DNA from a cohort of 124 European individuals, identified and described the minimal gut genome and metagenome in terms of functions, which is the foundation of many studies on gut microbiome function [26]. Arumugam et al. combined 22 sequenced faecal metagenomes of individuals from 4 countries with previously published datasets, identified 3 enterotypes that are geographically nonspecific, and multiple functional biomarkers for host properties including age, gender, and body mass index, which address the importance of analyzing gut microbiome function at metagenomic level [27].

Frank et al. used sequencing analysis of GI tissue samples from Crohn’s disease, ulcerative colitis, and healthy controls and studied microbiota differences between these groups. They demonstrated significant perturbations in the GI microbiotas of select IBD patients, which can be utilized as microbial markers for differentiation [2]. Sokol et al. introduced *F. prausnitzii* as an anti-inflammatory bacterium in Crohn’s disease population, and found that a lower proportion of *F. prausnitzii* on resected ileal Crohn disease mucosa was associated with endoscopic recurrence at 6 months. In vivo study on mice with induced colitis showed *F. prausnitzii* and its supernatant affected cytokine levels, markedly reduced the colitis severity, indicating the potential value of *F. prausnitzii* in Crohn’s disease treatment [12]. Sokol et al. utilized quantitative polymerase chain reaction to analyze fecal samples from subjects with active Crohn’s disease or ulcerative colitis, Crohn’s disease or ulcerative colitis in remission, infectious colitis, and healthy controls. The study showed underrepresentation of phylum Firmicutes, particularly *F. prausnitzii* in active IBD and infectious colitis patients, further addressing the important anti-inflammatory role of *F. prausnitzii* in gut inflammatory state [1]. Sartor gathered animal model evidence, clinical evidence and pathogenesis theories, extensively discussed on microbial influences on IBD and the underlying molecular mechanism, it is the most frequently cited review article in gastrointestinal microbiome [8].

Turnbaugh et al. used metagenomic analyses on mice gut microbiota model, and found that the obese microbiota is hyper-functional in harvesting energy. Transplantation of this obese microbiota resulted in increased total body fat in germ-free mice, indicating the importance of gut microbiome in the pathophysiology of obesity [3]. Turnbaugh et al. studied the faecal microbiome of adult female monozygotic and dizygotic twin pairs concordant for leanness or obesity, and their mothers, and revealed that gut microbiome is shared among family members with comparable degree of co-variation between monozygotic and dizygotic twin pairs, which adds evidence to help determine the relative contributions of host genetic vs environmental factors to gut microbial ecology. Obesity-associated gut microbial changes in diversity, community structure, and metabolic pathways were also identified, further yield association between obesity and deviation of core microbiome at a functional level [14].

The high frequency keywords including gut microbiota, IBD, irritable bowel syndrome, and probiotics in co-occurrence cluster analysis and co-cited reference cluster analysis (Figs. 2, 3b) indicate that gut microbiota and related digestive diseases, especially IBD, remained the hotspots in gut microbiome research. Analysis of top keywords, using burst detection, (Fig. 5) shows that dysbiosis, Clostridium difficile infection, and 16s rRNA attracted the most attention of peer researchers during the past 20 years, while obesity and *A. muciniphila* were among the new research foci since 2016. Research foci in gut microbiome seems to have shifted from IBD to obesity and liver diseases, from the traditional technique to 16s rRNA sequencing and metagenomics, and from basic science to clinical utilization.

During the past decades, a significant amount of research has been conducted to study the gut microbiota as a contributing factor to the pathogenesis of IBD. The pathogenesis of IBD has been proposed to be associated with disruption of mucosal homeostasis and dysregulation of hypersensitive response within gut-associated lymphoid tissue [2]. Crohn’s disease and ulcerative colitis are more likely to involve intestinal segments with higher intestinal bacteria concentration, alterations in the composition and metabolism of gut microbiota in IBD patients have been identified by molecular techniques [2, 28]. Studies have proposed that microbial diversity in active IBD patients is decreased, with depletion of commensal bacteria including Firmicutes and Bacteroidetes, and concomitant increase in Proteobacteria, Actinobacteria, and Enterobacteriaceae [2, 8]. Decrease in SCFA production by primarily Clostridia and Bacteroides that are depleted in IBD patients, and overproduction of hydrogen sulfide by overgrowing sulfate-reducing bacteria in ulcerative colitis patients can result in epithelial nutrition deficiency [8, 29, 30]. Recently, more and more evidence suggested that SCFA, particularly acetate, butyrate, and propionate, which were metabolized by gut bacteria from fiber-rich diet, has regulatory effect on intestinal immune response and anti-inflammatory effect thus alleviating autoimmune related diseases including IBD in animal models. Recently, butyrate, a bioactive SCFA, has also been shown to play a controversial role in tumorigenesis in the gut [31–33]. *F. prausnitzii* was identified as an anti-inflammatory commensal bacterium and might be a promising strategy in Crohn’s disease [12]. Antibiotics and probiotics have also been suggested to ameliorate some types of IBD by remediation of gut microbiota [34].

Obesity with its increasing prevalence and comorbidity worldwide has become a focus point of gut microbiome research during more recent years. Obesity has been shown to be associated with a significant decrease in the level of microbiota diversity, decreased proportion of Bacteroidetes, and increased proportion of Actinobacteria; while the proportion of Bacteroidetes increases with weight loss despite total caloric intake [14, 35]. Obesity-associated gut microbiota has been found to have an increased capacity for energy harvest; this was validated in animal studies by transplantation of lean and obese caecal microbiotas into germ-free mouse recipients [3]. These research findings also proposed that human obesity could be managed by manipulating gut microbiota, although further studies in human subjects are needed.

*Akkermansia muciniphila*, an abundant constituent of the human microbiota that resides in the mucus layer of the gut, is one of the promising targets in future generation probiotic research as it may play a role in the management of obesity and metabolic syndrome, inflammations, autoimmune diseases, and cancer. *A. muciniphila* is proposed to promote gut homeostasis and human health by inducing mucus production, innate immune signaling via Toll-like receptor [6], and expression of antibacterial peptide; causing microbiota remodeling; lower serum endotoxin; etc. [36, 37]. Studies suggested that *A. muciniphila* abundance is inversely correlated with obesity, metabolic syndrome, IBD, and acute appendicitis [38–41]. Recent study also suggests an association between *A. muciniphila* abundance and clinical response to PD-1-based immune check-point inhibitors [42]. Supplementation of *A. muciniphila* to mice gut has been shown to be protective against development of obesity, type 1 and type 2 diabetes, atherosclerosis, and poor response to the antitumor effects of PD-1 blockade [36, 38, 42–44]. Ongoing research is further investigating *A. muciniphila* as a therapeutic tool in the management of multiple diseases.

Intestinal microbiota-host interaction has been shown to play a role not only in gastrointestinal diseases, but also in extra-gastrointestinal diseases [45, 46]. Studies have shown correlation between intestinal microbiome and extra-gastrointestinal malignancy and its response to immunotherapy [47, 48], atherosclerotic cardiovascular disease. Wang et al. and Tang et al. [49, 50] psychiatric diseases including mood disorders, schizophrenia, and autism spectrum disorder [51–55], neurologic diseases including Alzheimer’s disease, Parkinson’s disease and multiple sclerosis [56–58], metabolic disorders including diabetes [59, 60], allergic/immunologic diseases including asthma, systemic lupus erythematosus, autoimmune arthritis, and inflammatory skin diseases [58, 61–63]. Targeting the gut microbiome dysbiosis to intervene in the underlying pathogenesis might be the new therapeutic approach for diseases of multiple systems.

Compared to traditional reviews, analysis based on Citespace provides a better insight of the evolving research foci and trends, but it comes with certain limitations. Similar words need be merged together during the analysis; even though only original articles were included in the majority of analysis, all article types were included during the co-cited reference analysis.

## Conclusions

There is no doubt that our understanding of gut microbiome has significantly advanced via bursts of high quality research occurring over the past 20 years. With the help of information visualization, we were able to identify research foci and overall trends in the field and offer gathered information to future researchers. We believe gut microbiota is associated with the pathogenesis of significantly more diseases than we currently know of. The emerging new therapeutic targets in gut microbiota would be the foci of future research.

## Abbreviations

16S rRNA: 16S ribosomal RNA
IBD: inflammatory bowel disease
WoScc: Web of Science Core Collection
SCFA: short-chain fatty acid
